# Sleepless latency of human cytomegalovirus

**DOI:** 10.1007/s00430-015-0401-6

**Published:** 2015-03-14

**Authors:** Emma Poole, John Sinclair

**Affiliations:** Department of Medicine, Box 157, University of Cambridge, Addenbrooke’s Hospital, Level 5 Laboratories Block, Hills Road, Cambridge, CB2 0QQ UK

**Keywords:** Latency, Human cytomegalovirus, Cell survival

## Abstract

As with all human herpesviruses, human cytomegalovirus (HCMV) persists for the lifetime of the host by establishing a latent infection, which is broken by periodic reactivation events. One site of HCMV latency is in the progenitor cells of the myeloid lineage such as CD34+ cells and their CD14+ derivatives. The development of experimental techniques to isolate and culture these primary cells in vitro is enabling detailed analysis of the events that occur during virus latency and reactivation. Ex vivo differentiation of latently infected primary myeloid cells to dendritic cells and macrophages results in the reactivation of latent virus and provides model systems in which to analyse the viral and cellular functions involved in latent carriage and reactivation. Such analyses have shown that, in contrast to primary lytic infection or reactivation which is characterised by a regulated cascade of expression of all viral genes, latent infection is associated with a much more restricted viral transcription programme with expression of only a small number of viral genes. Additionally, concomitant changes in the expression of cellular miRNAs and cellular proteins occur, and this includes changes in the expression of a number of secreted cellular proteins and intracellular anti-apoptotic proteins, which all have profound effects on the latently infected cells. In this review, we concentrate on the effects of one of the latency-associated viral proteins, LAcmvIL-10, and describe how it causes a decrease in the cellular miRNA, hsa-miR-92a, and a concomitant upregulation of the GATA2 myeloid transcription factor, which, in turn, drives the expression of cellular IL-10. Taken together, we argue that HCMV latency, rather than a period of viral quiescence, is associated with the virally driven manipulation of host cell functions, perhaps every bit as complex as lytic infection. A full understanding of these changes in cellular and viral gene expression during latent infection could have far-reaching implications for therapeutic intervention.

## Introduction

Human cytomegalovirus (HCMV) is typical of human herpesviruses in that it has both a latent and a lytic phase of its life cycle, which are bridged by periodic reactivation events. In the immune competent, primary infection is rarely symptomatic, and following the establishment of latency, the virus maintains a persistent infection effectively controlled by the immune system. It is now established that, in vivo, one site of HCMV latency is in bone marrow resident CD34+ myeloid progenitor cells as well as in their derivative CD14+ monocytes present in peripheral blood. Whilst there is little consensus, likely due to differences in cell types analysed and models of latency used, in the spectrum of viral genes expressed during latent infection, it is clear that latency is associated with a much restricted virus gene transcription programme and, in general, an absence of expression of viral major lytic genes [1–5]. This is also true for cytomegalovirus infection in other species, although the mechanisms by which latency is established vary. A number of studies have demonstrated that murine cytomegalovirus (MCMV) establishes latency, which suggests that some parallels may be drawn between the two species [6, 7]. This inhibition of lytic gene expression during latency is likely affected through repressive chromatin marks around the promoter of the viral major immediate early promoter (MIEP). Following differentiation of progenitor myeloid cells into terminally differentiated dendritic cells or macrophages, however, this repressive chromatin structure around the MIEP is relieved, resulting in changes in post-translational modifications of histones around the MIEP associated with transcriptional activation and concomitant induction of viral lytic immediately early (IE) gene expression [5, 8–12]. These data imply that reactivation routinely occurs in vivo, but this is sub-clinical due to robust host immune responses and is supported by the recent observations that macrophages and DCs, in vivo, are sites of virus reactivation in the healthy carrier [13, 14].

## Main text

### There are numerous effects on the host cell during HCMV latency

**Fig. 1:**
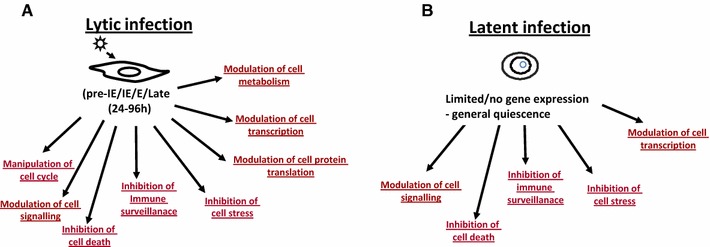
Manipulation of host cell functions during latent HCMV infection, perhaps every bit as complex as lytic infection. Infection of cells results in a wide range of changes to the infected cell. During lytic infection (**a**), there are reported changes to the modulation of cell metabolism, transcription, translation, cell cycle, cell signalling as well as the inhibition of immune surveillance, cell stress, and cell death [15–25]. Similarly, during latent infection (**b**), there are reported changes to the manipulation of host cell transcription and cell signalling and, again, the inhibition of host immune surveillance, cell stress, and cell death [4, 5, 31–36]

**Fig. 2:**
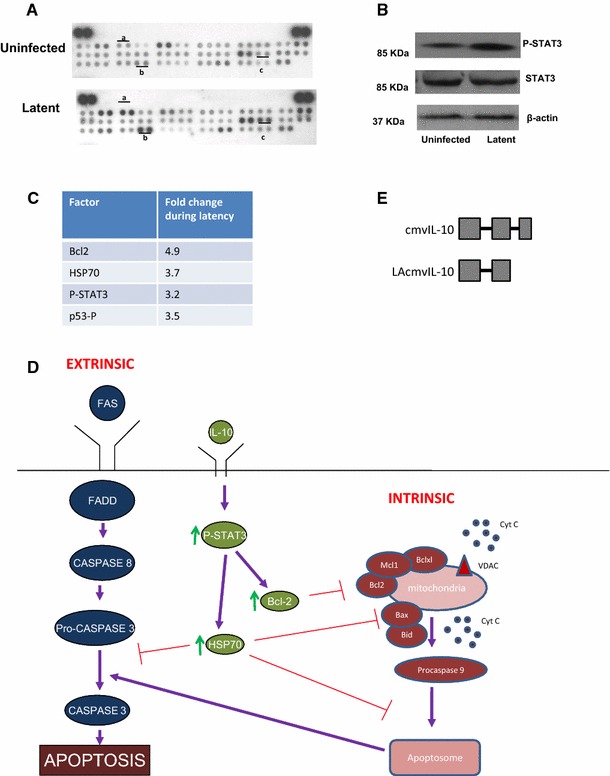
A number of pro- and anti-apoptotic factors in the FAS signalling pathway are altered during HCMV latency. Either CD34+ cells were uninfected or HCMV latency was established for 10 days, and the cells were harvested for protein analysis (**a**–**d**). Relative levels of proteins involved in FAS-mediated and IL-10 signalling were analysed by apoptosis array (R&D systems) (**a**) and *highlighted* are Aa (Bcl2), Ab (p53 phospho-serine-15), and Ac (HSP70). Alternatively, cells were harvested for Western blot analysis of total and phosphorylated STAT3 (antibodies from cell signalling) relative to actin loading control (antibody from Abcam) (**b**) and quantified by densitometry. Data are represented as fold change during latency from representative Western blots (**c**). The data are summarised in context with the literature in (**d**). Extrinsic FAS-mediated apoptosis involves FADD, caspase 8, pro-caspase 3, and caspase 3 and leads to apoptosis [81, 82]. Intrinsic mitochondrial-mediated apoptosis involves Bax, Bid, voltage-dependent anion channel (VDAC), cytochrome C (cyt c), pro-caspase 9, and the apoptome [83, 84]. Additionally, anti-apoptotic IL-10 signalling can involve STAT3 phosphorylation, Bcl2, and HSP70 [59, 61, 62]. These anti-apoptotic factors are all positively regulated during HCMV latency. Finally, the two isoforms of virally induced IL-10, cmvIL-10 and LAcmvIL-10, are shown in (**e**), where *grey boxes* represent exons. LAcmvIL-10 is generated from alternative splicing, which does not express exon 3

It is well established that the numerous HCMV-encoded genes expressed during lytic infection act in concert to exert profound effects on the infected cell, resulting in the modulation of a wide range of cell functions and their downstream effects. This includes modulation of cell metabolism, transcription, translation, cell cycle, cell signalling as well as the inhibition of immune surveillance, cell stress, and cell death [15–30] (Fig. 1a). However, despite a much restricted transcription profile, a number of studies have also shown that latent infection is associated with a profound manipulation of host cell transcription and cell signalling and, again, the inhibition of host immune surveillance, cell stress, and cell death [4, 5, 31–36] (Fig. 1b). Thus, far from being silent, latent infection with HCMV also results in the viral-driven orchestration of cellular gene expression and cell functions, likely, to optimise the cell for latent carriage and reactivation. For example, a number of changes in total cellular mRNAs have been shown to occur upon latent during experimentally latent infection of granulocyte macrophage progenitors (GMPs) [37, 38] resulting in changes in MHC class II expression and secreted monocyte chemoattractant protein-1 (MCP-1) also known as chemokine C–C motif ligand-2 (CCL2) [39]. Consistent with this, latent infection of myeloid progenitors also results in the regulation of MCP-1 as well as a large number of other secreted cell proteins [32]. In addition to the regulation of secreted proteins during latency, an apoptome array shows that there are also a number of changes in levels of anti-apoptotic proteins during latent infection of CD34+ cells with HCMV (Fig. 2).

Given the relative paucity of viral genes expressed during HCMV latency [1–5] compared to lytic infection, it may initially seem surprising that such profound changes in the cell result from latent infection. However, another level of regulation of gene expression is via microRNAs (miRNAs). These are highly conserved small (approximately 21 nucleotides in length) RNA molecules encoded in the genomes of plants and animals, which generally regulate the expression of genes by binding to the 3′-untranslated regions (3′-UTR) of specific mRNAs.

Although the first published description of an miRNA was in 1993 [40], the understanding of the functions of many of these small non-coding RNA molecules is still being elucidated and is complicated by the fact that each miRNA is thought to be able to regulate multiple genes. This, coupled with the fact that there are hundreds of miRNAs transcribed in the cells of higher eukaryotes [41–43], reflects the enormous complexity in levels of regulation of gene expression afforded by miRNAs. Various lines of research suggest that miRNAs may act as key regulators of processes as diverse as early development [44], cell proliferation and cell death [45], apoptosis, and fat metabolism [46], as well as cell differentiation [47–49]. There is also evidence to suggest that miRNA expression is involved in the pathogenesis of a number of diseases, including cancer [50, 51] and viral infection [31, 52–55].

It is interesting, therefore, that during HCMV latency, there are changes to a number of cellular miRNAs ([31] and Table 1). Additionally, HCMV itself encodes a number of miRNAs. Consequently, it is possible that many of the changes in the latency-associated secretome as well as the observed changes in anti-apoptotic proteins during latent infection is due, at least in part, to the HCMV-mediated regulation of cellular miRNAs [31] as well as, possibly, expression of a number of viral miRNAs [56].

**Table 1:** HCMV-induced latency leads to changes in a number of cellular miRNAs

MicroRNA	Fold change during latency compared to mock
hsa-miR-let-7a	−2.5
hsa-miR-let-7b	−3.7
hsa-miR-206	−2
hsa-miR-296 3p	−2.6
hsa-miR-297	−2.9
hsa-miR-32*	−2
hsa-miR-608	−2.4
hsa-miR-92a	−2.5

Following the establishment of latency for 10 days in CD34+ cells, the cells were harvested for miRNA analysis (nCode, Invitrogen), and data are presented as fold change over mock infected cells with probability values

### Downregulation of a cellular miRNA can lead to upregulation of cellular proteins during latency

One of the cellular miRNAs which is known to be downregulated during latent infection of CD34+ progenitor cells is hsa-miR-92a ([31] and Table 1). Predictive algorithms and biochemical analysis have shown that this miRNA can target the myeloid transcription factor GATA2, and as predicted, during experimental HCMV latency, there is an increase in levels of this myeloid cellular transcription factor [31, 34].

**Fig. 3:**
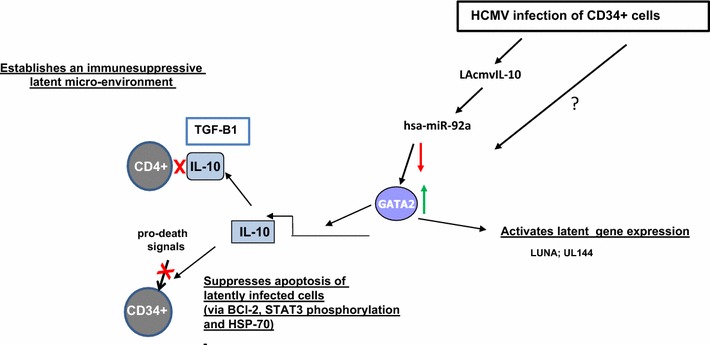
Myeloid transcription factor GATA2 plays multiple roles during HCMV latency. Following the establishment of latency in CD34+ cells for 10 days, there is an induction of cellular hsa-miRNA-92a via LAcmvIL-10 [31, 79]. This leads to a direct upregulation of the cellular transcription factor GATA2 [34]. GATA2 can drive the transcription of the latency-associated viral products LUNA and UL144 [34, 85] as well as driving transcription of the cellular cytokine gene IL-10 [31]. Whether other mechanisms for the upregulation of GATA2 during HCMV latency are also induced is not yet known. IL-10 serves to create an immune suppressive environment [32] as well as to lead to pro-life signalling to the latently infected cell [31]. IL-10 leads to STAT3 phosphorylation and signals to anti-apoptotic factors Bcl2 and HSP70 (see Fig. 2)

GATA2 is a cellular transcription factor known to be important in the proliferation, lineage commitment, and survival of haematopoietic progenitor cells [57–60], and the virus targets this important myeloid transcription factor for a number of now well-established reasons. For instance, GATA2 has also been found to regulate the transcription of the latency-associated viral gene product UL144 and may also play a role in the expression of other latency-associated viral gene products. GATA2 not only regulates viral genes but is also known to regulate the expression of a number of cellular genes, including IL-10. Consistent with this, increases in cellular IL-10 (cIL-10) in the secretome of latently infected CD34+ cells have been shown to occur. Detailed analysis of the mechanism by which latency-associated changes in hsa-miR-92a were linked to GATA2 expression and subsequent regulation of cIL-10 came from studies in KG1 cells, a CD34+ cell line which can be manipulated by transfection and recapitulate some aspects of HCMV latent infection, namely the expression of latency-associated genes such as UL138 in the absence of lytic immediate early gene expression [31]. In these cells, transfection of an antagomir to hsa-miR-92a led to increased GATA2 mRNA expression [31] and increased cIL-10. Importantly, this induction of cIL-10 by hsa-miR-92a antagomir did not occur if GATA2 was depleted by RNAi [31]. Taken together, these studies showed that latency-associated changes in hsa-miR-92a result in increased GATA2, which drives the expression of cIL-10 during latent infection (Fig. 3).

### Cellular IL-10 is important for the maintenance of latency and immune evasion

Cellular IL-10 is a secreted cytokine known to have immune modulatory properties as well as having pro-life effects on myeloid progenitors, such as CD34+ cells, by driving the expression of the anti-apoptotic factor Bcl2 (Fig. 2a, c, d and [31]). During HCMV latency, cIL-10 is upregulated, and for this reason, the specific functions of cIL-10 in the latent secretome have been analysed [32]. For example, consistent with this observation that cIL-10 is known to play an anti-apoptotic role in CD34+ cells (Fig. 2d), [31, 61], antibody depletion of latency-associated secretion of cIL-10 from the supernatants of latently infected cells results in increased cell death and loss of latent viral genome carriage [31]. Additionally, latency-associated secretion of cIL-10, together with virally induced increases in cellular TGF-beta, results in the establishment of an immune suppressive microenvironment around latently infected cells. This, in turn, inhibits CD4+ cytotoxic T-cell effector functions and thereby suppresses host immune surveillance of the latently infected cell [32].

We know that latent infection of primary CD34+ progenitor cells by HCMV results in their increased survival in the face of pro-apoptotic signals, and this, at least in part, appears to involve the known latency-associated increase in the expression of cIL-10 [31, 59]. However, how cIL-10 mediated this protection was unclear, but the changes in the latent apoptome would suggest that the cIL-10 upregulated during latency modulates cIL-10-mediated suppression of extrinsic and intrinsic pro-apoptotic signals (Fig. 2d).

Engagement of the cIL-10 receptor by cIL-10 is known to induce signalling via STAT3 phosphorylation. This results in positive autoregulation of cIL-10 expression as well as expression of intrinsic death-signalling pathway inhibitors such as Bcl2 [31, 59] and HSP70 [62]. Consistent with this, and the known increase in cIL-10 during HCMV latent infection of CD34+ cells [32], latently infected CD34+ cells also showed extensive increases in STAT3 phosphorylation (Fig. 2b, c) and concomitant increases in the expression of Bcl2 and HSP70 (Fig. 2a, c). HSP70 plays a role in the negative regulation of the intrinsic pathway due to the ability to target pro-caspase 3 [63, 64], present in the FAS-mediated signalling pathway. Thus, during latency, the FAS-mediated signalling pathway is targeted at different stages of FAS-mediated apoptosis via extrinsic apoptosis signalling (Fig. 2d). Interestingly, HSP70 also plays a significant role as a potent inhibitor of the formation of the mitochondrial apoptome [65] and the intrinsic pathway of programmed cell death (Fig. 2d).

Not all of the changes in proteins we have identified during HCMV latency are in pro-life factors. Figure 2a, c shows that there was a 3.5-fold increase in levels of p53 phosphorylated at serine 15. Ser 15-phosphorylated p53 is known to have pro-apoptotic properties as it is able to upregulate the transcription of the pro-apoptotic factor Bax and concomitantly decrease the expression of anti-apoptotic factor Bcl2 [66]. However, the expression of Bcl2 clearly increased during HCMV latency in our studies (see Fig. 2a, c and [31]) despite elevated levels of phosphorylated p53. Consequently, our view is that other functions associated with latent infection also counter the transcriptional regulation of Bcl2 by p53. One possibility is that HSP70 increased during latent infection and can act to stimulate the expression of Bcl2 via AKT [67]. Therefore, the expression of HSP70 may be strong enough to overcome the repressive effects of phosphorylated p53. Similarly, formation of pro-apoptotic Bax homodimers can be prevented by HSP70 [65]. Therefore, it is likely that HSP70 works at multiple levels to help check and balance the levels of anti-apoptotic factors in the latently infected cell, although this needs to be formally addressed.

### Latency-associated viral IL-10 can cause downregulation of the cellular miRNA hsa-miR-92a

It appears, then, that the changes in cellular miRNA expression resulting from latent infection can have important downstream effects on both intrinsic cell survival and host immune evasion. However, until recently, the mechanism by which HCMV latent infection caused such changes in cellular miRNAs expression was far from clear. There are a number of viral genes expressed during latency, which could potentially affect cellular miRNA expression, although the latency-associated functions of many of these latency-associated genes are only just beginning to be unravelled.

One of these is a viral cIL-10 homologue, known as LAcmvIL-10, encoded by the UL111A gene. It is interesting that although latent HCMV infection robustly induces the expression of cIL-10, as discussed above, the virus also expresses LAcmvIL-10 during this life cycle. However, HCMV is not unique amongst the herpesviruses in encoding a cIL-10 homologue [68]. However, HCMV actually encodes two IL-10 homologues. These two isoforms of virus-encoded IL-10 are generated by alternative splicing from the viral UL111A gene as depicted graphically in Fig. 2e. One of these is a protein of 175 amino acids, termed cmvIL-10, which is expressed during lytic infection and has the expression kinetics of a late gene. The second isoform, predicted to consist of 139 amino acids and termed LAcmvIL-10, has a C-terminal truncation and is expressed during both lytic and latent infection [68–70]. The encoding of a cIL-10 homologue is not unique to HCMV. An IL-10 homologue encoded by the UL111A open reading frame (ORF) has also been identified in rhesus macaque CMV (RhCMV). Although it has a slightly different gene structure than cmvIL-10, like cmvIL-10, it shows low amino acid identity to host cIL-10 [69].

The full-length cmvIL-10 gene shares 27 % amino acid homology with cIL-10 [71] and has a number of functions in common with cIL-10: it forms homodimers and binds the cIL-10 receptor [69, 72]; it triggers STAT3 phosphorylation and activation of the JAK/STAT signalling pathway [73, 74]; it signals via the phosphoinositide-3-kinase pathway, contributing to cytokine suppression [74–76] and cIL-10-positive autoregulation [77]; and it shares the ability of cIL-10 to prevent NF-κB activity via inhibition of IKK [75, 78].

In contrast, LAcmvIL-10 appears quite dissimilar to cIL-10 and cmvIL-10. Although, like cIL-10 and cmvIL-10, it can downregulate major histocompatibility complex (MHC) class II in latently infected GMPs [76], either it does not signal through the IL-10 receptor (IL-10R) or it engages the receptor in a different way than cIL-10 and cmvIL-10. Therefore, the mode of action and function during HCMV latency is uncertain.

Interestingly, analysis of viruses lacking the UL111A gene locus was found to be impaired in their ability to induce cIL-10 upon latent infection [79]. Similarly, consistent with these observations, viruses lacking UL111A did not downregulate cellular hsa-miR-92a. Furthermore, recombinant LAcmvIL-10 was found both to induce cIL-10 and to cause the downregulation of hsa-miR-92a [79].

Taken together, then it appears that expression of LAcmvIL-10 during latent infection results in downregulation of cellular hsa-miR-92a, which in turn leads to the upregulation of the myeloid transcription factor GATA2. This increase in GATA2 then drives the expression of cIL-10, which inhibits intrinsic cell death signals and aids immune evasion of the latently infected cell (Fig. 3).

It is also worth emphasising that GATA2 is known to be involved in the hematopoiesis and myeloid cell differentiation [80]. The extent to which latent infection of CD34+ cells, in itself, drives the latently infected progenitor cell down the myeloid lineage, rather than the lymphocyte lineage, is unclear. However, this could, in part, explain the fact that latent viral genomes have not been detected in T and B cells even though these presumably derived from the same CD34+ progenitor cells giving rise to cells of the myeloid lineage.

### Future perspectives

The ramifications of latency-associated changes in a number of other cellular miRNAs that have been identified during latent (Table 1 and [31]) infection are unclear. Similarly, latent infection also results in changes in a number of other secreted cellular proteins [32], and the effects of these changes on the latently infected cell will be enlightening. Regardless, it is now clear that latent infection imparts on the latently infected cells a plethora of phenotypic changes through an orchestrated manipulation of cell gene expression and cell functions. These are likely necessary for efficient carriage and reactivation of latent viral genomes, but they may also provide an ‘Achilles heel’ to allow the development of novel therapeutics to target and clear latent infection, at least in some clinical settings.
